# The inflammatory signalling mediator TAK1 mediates lymphocyte recruitment to lipopolysaccharide-activated murine mesenchymal stem cells through interleukin-6

**DOI:** 10.1007/s11010-021-04180-8

**Published:** 2021-05-29

**Authors:** Beatrice Oelze, Kirsten Elger, Patrik Schadzek, Laura Burmeister, Anika Hamm, Sandra Laggies, Virginia Seiffart, Gerhard Gross, Andrea Hoffmann

**Affiliations:** 1 Helmholtz Centre for Infection Research (HZI), Inhoffenstr. 7, 38124 Braunschweig, Germany; 2 Hannover Medical School, Department of Orthopaedic Surgery, Graded Implants and Regenerative Strategies OE 8893, Stadtfelddamm 34, 30625 Hannover, Germany; 3 Hannover Medical School, Lower Saxony Centre for Biomedical Engineering, Implant Research and Development (NIFE), Stadtfelddamm 34, 30625 Hannover, Germany

**Keywords:** Promiscuous function of TAK1, Inflammation, Infection, Differentiation

## Abstract

As a response to pro-inflammatory signals mesenchymal stem cells (MSCs) secrete agents and factors leading to lymphocyte recruitment, counteracting inflammation, and stimulating immunosuppression. On a molecular level, the signalling mediator TGF-β-activated kinase 1 (TAK1) is activated by many pro-inflammatory signals, plays a critical role in inflammation and regulates innate and adaptive immune responses as well. While the role of TAK1 as a signalling factor promoting inflammation is well documented, we also considered a role for TAK1 in anti-inflammatory actions exerted by activated MSCs. We, therefore, investigated the capacity of lipopolysaccharide (LPS)-treated murine MSCs with lentivirally modulated TAK1 expression levels to recruit lymphocytes. TAK1 downregulated by lentiviral vectors expressing TAK1 shRNA in murine MSCs interfered with the capacity of murine MSCs to chemoattract lymphocytes, indeed. Analysing a pool of 84 secreted factors we found that among 26 secreted cytokines/factors TAK1 regulated expression of one cytokine in LPS-activated murine MSCs in particular: interleukin-6 (IL-6). IL-6 in LPS-treated MSCs was responsible for lymphocyte recruitment as substantiated by neutralizing antibodies. Our studies, therefore, suggest that in LPS-treated murine MSCs the inflammatory signalling mediator TAK1 may exert anti-inflammatory properties via IL-6.

**Supplementary Information:**

The online version contains supplementary material available at 10.1007/s11010-021-04180-8.

## Introduction

Mesenchymal stem cells (MSCs) are mesodermal progenitor cells which possess a wide range of differentiation capacities. These multipotent stem cells are able to differentiate into a number of mesenchymal cell types, like osteoblasts, chondrocytes and adipocytes, into myocytes and, under specific conditions, seem also to generate ectodermal and endodermal cell types, like neuronal cell types or β-pancreatic islets cells, respectively [1–4]. MSCs exert immunosuppressive effects in both the human and murine system, in vitro and in vivo. Numerous studies have established that MSCs lack co-stimulatory molecules, such as CD80, CD86 and human leukocyte antigen (HLA); thus, they fail to activate allo- or xenogeneic immune cells due to the lack of immunogenicity [5]. MSCs may modify the innate and adaptive immune response, interfere with T cell proliferation [6–8], reduce the maturation and antigen presentation of dendritic cells [9], modulate B cell function [10] or block natural killer cell activation [11].

The mechanisms of MSC-dependent immunosuppression are not entirely elucidated but seem mainly to depend on cell–cell interaction and the secretion of soluble factors [12]. MSCs stimulated by pro-inflammatory cytokines and agents, such as IFN-γ, TNF-α, IL-1α, IL-1β or lipopolysaccharide (LPS), secrete soluble factors some of which have been proposed to be responsible for the immunosuppressive effect of MSCs, in particular, indoleamine-pyrrole 2,3-dioxygenase (IDO), prostaglandin E2 (PGE2), nitric oxide (NO), transforming growth factor-β1 (TGF-β1), hepatocyte growth factor (HGF) cyclooxygenase 2 and TGF-β1 [12–17]. Additional factors have lately been included as important for the immunosuppressive role of MSCs, such as the leukaemia inhibitory factor [18], galectins [19] and interleukin-6 (IL-6) [20, 21]. Several studies showed that MSC administration in vivo prevented the development of graft-*versus*-host disease and suppressed delayed-type hypersensitivity [7, 12]. Consequently, MSCs in the meantime are widely used in clinical trials due to their immunosuppressive and tissue regeneration capacities.

A major signalling mediator involved in innate and acquired immune responses is transforming growth factor- β (TGF- β)-activated kinase 1 (TAK1, also known as MAP3K7). Originally, this important inflammatory signalling factor was identified as a kinase responsive to TGF-β and BMPs [22]. Subsequent studies showed that, in addition, TAK1 integrates pro-inflammatory signals mediated from cytokine-, Toll-like- (TLR), T and B cell receptors as well [23, 24]. Moreover, TAK1 is also involved in stress–response signalling [25, 26]. To exert its biological activity, TAK1 interacts with activator proteins which are called TAK1 binding proteins (TAB1, TAB2, TAB3) [27, 28]. TAK1 regulates the activity of two major transcription factors involved in inflammation, nuclear factor κB (NFκB) and transcription factor activator protein-1 (AP-1) [24, 29]. In a murine collagen-induced arthritis model we demonstrated that in vivo downregulation of TAK1 efficiently alleviates inflammation, interferes with different inflammatory signalling pathways and decreases the frequency of inflammatory Th1 and Th17 cells emphasizing the importance of TAK1 also in chronic inflammatory disorders [30].

While the role of the signalling mediator TAK1 as a major factor promoting inflammatory pathways is well documented, we developed the hypothesis that TAK1 might also be involved in the establishment of anti-inflammatory properties of MSCs subsequent to their activation by inflammatory agents or cytokines. We, therefore, investigated the capacity of lipopolysaccharide (LPS)-treated MSCs exhibiting lentivirally modulated TAK1 expression levels to recruit lymphocytes. LPS is a major structural constituent of the outer membrane of Gram-negative bacteria and triggers the pattern recognition receptor Toll-like receptor 4 (TLR4) to initiate the downstream signalling cascade via TAK1, reviewed in [31]. We found that among 84 cytokines IL-6 was the only LPS-stimulated and secreted cytokine in murine MSCs (mMSCs) regulated in a TAK1-dependent fashion. IL-6 was responsible for recruitment of lymphocytes to LPS-activated mMSCs as substantiated by neutralizing antibodies indicating that in MSCs the inflammatory signalling mediator TAK1 may be involved in the establishment of anti-inflammatory and/or immunosuppressive properties, indeed.

## Materials and methods

### Chemicals and reagents

The lipopolysaccharide (LPS) preparation was from Escherichia coli O111:B4 (Invivogen tlrl-3pelps, 5E + 06EU/ml). Buffer substances were from standard laboratory suppliers, including Carl Roth (Karlsruhe, Germany) and Sigma (Taufkirchen, Germany). The suppliers of specific reagents are indicated in the respective methods section.

### HEK293T cell line for lentivirus generation

Human embryonic kidney 293 T (HEK293T; ATCC® CRL-3216™) cells were cultivated in Dulbecco´s modified Eagle´s medium (DMEM) with 4.5 g/l glucose (Biochrom F0445) containing 10% foetal bovine serum (Thermo Fisher/ Invitrogen, Karlsruhe, Germany), 100 U/ml penicillin (Sigma) and 100 µg/ml streptomycin (Biochrom A2213, Berlin, Germany). All cell cultures were maintained at 37 °C and with 5% CO_2_.

### Mice

C57BL/6 mice were from Harlan Laboratories. For preparation of bone marrow and spleen, they were anaesthetized by isoflurane and killed by cervical dislocation. Dead mice were soaked in 70% ethanol for 5 to 10 min for disinfection.

### Splenocyte isolation

The spleen was prepared and stored in a 6-well plate in phosphate-buffered saline (PBS, without Ca^2+^, without Mg^2+^). It was cut into small pieces with a scalpel and then pressed with the back of a 2 ml syringe plunger until only the turbid empty sheath was left. The resultant cell suspension was filtered through a nylon filter (100 µm mesh size: BD Bioscience) into a conical 50-ml vessel. The 20 ml of suspension (per spleen) was centrifuged for 5 min at 250 × g at 4 °C. After removal of the supernatant the cell pellet was resuspended in 5 ml of ACK lysis buffer (155 mM NH_4_Cl/ 10 mM KHCO_3_/ 0.1 mM EDTA) in order to lyse erythrocytes, incubated for 3 min at room temperature and centrifuged as before. The supernatant was discarded, the pellet was washed with PBS and recentrifuged as before. The cell pellet was suspended in 10 ml splenocyte medium (88% DMEM with 1 g/l glucose: Biochrom F 0415, 10% foetal calf serum (Thermo Fisher/ Invitrogen), 1% 100 × penicillin/ streptomycin mixture (Biochrom A2213), 2 mM glutamine (Biochrom K0283), 10 mM HEPES (Biochrom L1613), 500 µM β-mercaptoethanol (Invitrogen 31,350–010), 1 mM sodium pyruvate (Invitrogen 11,360–039) and 1% MEM non-essential amino acids (Invitrogen 11,140–035)). The cell concentration (diluted 1: 4000) was determined with a Casy® Cell Counter (Schärfe System GmbH, Reutlingen, Germany).

### MSC isolation and propagation

The entire procedure was conducted under sterile conditions in a laminar flow cabinet. From the hind legs of the mice, femur and tibia were dissected and stored in wells of a 6-well plate with phosphate-buffered saline (PBS, without Ca^2+^, without Mg^2+^). After removal of the proximal and distal ends of the bones they were stored in another well with medium (88% DMEM with 1 g/l glucose, 10% foetal calf serum, 1% 100 × penicillin/ streptomycin mixture, 2 mM glutamine). The bone marrow was flushed from the bones with medium, homogenized by a 23-G needle and the resultant cell suspension was transferred into a conical 50-ml tube. The suspension was filtered through a nylon filter (100 µm mesh size: BD Biosciences, Heidelberg, Germany) into a fresh conical 50-ml vessel. The suspension was centrifuged form 5 min at 450 × g at room temperature. After removal of the supernatant the cell pellet was resuspended in 5 ml mMSC medium (the medium quoted above supplemented with 2 ng/ml FGF2 (Peprotech 100-18B, Hamburg, Germany)) and material isolated from one mouse was seeded into one T25 Roux culture flask. Medium was changed after 4 h. The day after cell isolation medium changes were performed in the morning and afternoon, again after another 2 or 3 days. Outgrowing colonies of plastic-adherent cells were harvested by trypsin–EDTA (0.05%/ 0.02% (w/v) in 1 × PBS: Biochrom L2153) before reaching confluence and subcultured at a density of 1,000 to 5,000 cells/cm^2^ in mMSC medium. Macrophages that are stronger plastic adherent than mMSCs were lost during passaging. For all experiments, cells were used in passage 9 or later (corresponding to about 11 weeks of cultivation after isolation). At this stage, the culture was morphologically homogeneous. MSC characteristics were confirmed by flow cytometric analysis of cell surface molecules and by in vitro differentiation into the osteogenic, adipogenic and chondrogenic lineage.

### Flow cytometric analysis

The cell surface expression of selected antigens was demonstrated by flow cytometric analysis. mMSCs were detached from the culture vessel with trypsin–EDTA. The reaction was stopped with 2% foetal calf serum in PBS (= FACS buffer). After cell counting the suspension was centrifuged at 200 × g for 5 min at 4 °C. The cell pellet was resuspended in FACS buffer to give 2.5 × 10^6^ cells/ml. Aliquots of 100 µl were centrifuged as before; the cell pellets were incubated with the respective labelled primary antibody (recombinant human monoclonal antibodies against the selected antigens), isotype control or FACS buffer (= NTC) (all diluted 1: 50 in 100 µl) for 15 min at 4 °C. The samples were centrifuged, washed with 500 µl FACS buffer and after centrifugation suspended in 500 µl FACS buffer. The samples were analysed in a MACSQuant® Analyzer 10 (Miltenyi Biotech). All antibodies were purchased from Miltenyi Biotech: human anti-mouse CD34 (FITC REA383; #130–117-775); human anti-mouse CD44 (APC-Vio770 REA664; #130–118-695); human anti-mouse CD45 (Vioblue REA737; #130–110-802); human anti-mouse CD73 (APC-Vio770 REA778; #130–111-520); human anti-mouse CD90.2 (APC REA1167; #130–120-898); human anti-mouse CD105 (FITC REA1058; #130–118-173); human anti-mouse Sca-1 (APC REA422; #130–123-848); isotype control (S) Vioblue (REA293; #130–104-609); isotype control (S) APC (REA293; #130–104-614) and isotype control (S) APC-Vio770 (REA293; #130–104-618). For data analysis, Flowjo™ software (Becton Dickinson) was used.

### Differentiation experiments

For osteogenic and adipogenic differentiation, mMSCs were seeded at 5,000 cells/cm^2^, respectively, into T25 Roux flasks in mMSC medium. At confluence (arbitrarily termed day 0), medium was replaced by the respective induction medium. The osteogenic differentiation medium consisted of 87% DMEM (Biochrom FG 0415 with 1 g/l glucose), 10% foetal calf serum, 20 mM HEPES, 1% 100 × penicillin/streptomycin, 10 mM β-glycerophosphate, 50 µg/ml ascorbic acid and 100 nM dexamethasone. The adipogenic differentiation medium consisted of 77% DMEM (Biochrom FG 0435 with 4.5 g/l glucose), 20% foetal calf serum, 20 mM HEPES, 1% 100 × penicillin/streptomycin, 500 µM 3-isobutyl-1-methylxanthine, 60 µM indomethacin and 1 µM dexamethasone. Medium changes were performed twice weekly for up to 21 days. At the end of the cultivation, cells were fixed with 3% (w/v) paraformaldehyde in PBS for 30 min at 4 °C followed by 3 × washing with PBS and storage in PBS. For staining, samples were washed 3 × with water (Millipore quality). Alkaline phosphatase activity in osteoblasts was visualized by cellular staining with 5-bromo-4-chloro-3-indolyl phosphate/ nitro blue tetrazolium (Sigma FAST BCIP/NBT) as described in the manufacturer’s protocol. Adipocytes demonstrate lipid droplets which can be stained by lipophilic dyes. Here, 0.5% (w/v) Oil Red O in 60% (v/v) isopropanol was used. After staining, samples were washed with Millipore quality water mounted with Kaiser´s glycerol gelatin, and documented microscopically. For chondrogenic differentiation, a cell suspension was centrifuged at 200 × g for 5 min at room temperature. The pellet was resuspended in 5 ml incomplete chondrogenic differentiation medium (97% DMEM (Biochrom FG 0435 with 4.5 g/l glucose), 20 mM HEPES, 1% 100 × penicillin/streptomycin, 100 nM dexamethasone, 170 µM ascorbate-2-phosphate, 1 mM sodium pyruvate, 350 µM proline, 6.25 µg/ml human recombinant insulin, 6.25 µg/ml human natural transferrin, 6.25 ng/ml selenious acid, 5.35 µg/ml linoleic acid and 1.25 mg/ml bovine serum albumin (ITS). After centrifugation as before 125,000 cells were resuspended in 500 µl complete chondrogenic differentiation medium (10 ng/ml TGF-β_3_ added to the incomplete medium). Medium changes in the pellet cultures were performed every other day. At the end of the cultivation period the pellets were fixed with Histofix (Roth) for 48 h at room temperature. Afterwards the pellets were stored in 70% ethanol. The pellets were embedded in paraffin, cut into 1 µm thick sections and stained. Proteoglycan-secreting chondrocytes were identified by staining with Alcian Blue 8GX (Sigma) and Safranin Orange. *Alcian Blue Staining:* The slides were stained with a 0.5% (w/v in 0.1 M HCl) Alcian Blue 8GX (Carl Roth) solution for 30 min at room temperature. They were washed several times with purified water until no more discolouration occurred. Before photographic documentation, slides were air-dried. *Safranin Orange Staining:* The slides were stained with a 0.1% (w/v in water) Safranin Orange (Carl Roth) solution for 15 min at room temperature, washed several times with purified water until no more discolouration occurred and documented microscopically.

### Construction of lentiviral targeting construct for knockdown of TAK1 (shTAK1) and the control (shCTR)

Vectors were constructed by using standard cloning procedures. The following sequence was used for generation of the shTAK1 hairpin: 5´-TGGCGTATCTTACACTGGATTCAAGAGATCCAGTGTAAGATACGCCA (underlined: sense, bold: stem loop, normal print: antisense). This sequence has successfully been used before in previous studies of others and our own group [30, 32]. It was cloned into pSUPER [33] under the control of its H1 promoter. The shCTR sequence was as detailed in [34].The correct nucleotide sequences were confirmed by DNA sequencing of both strands with the ABI Prism 310 capillary sequencer (BigDye Terminator Cycle Sequencing Ready Reaction Mix v.1.1; Applied Biosystems, Darmstadt, Germany). Subsequently, the H1 promoter-shRNA cassettes were excised from pSUPER by *Sma* I and *Hinc* II restriction enzymes and transferred into the vector pSR by blunt-end cloning into its dephosphorylated *Sna* BI site. The pSR vector with red fluorescent protein (RFP) under control of a SFFV (spleen focus-forming virus) promoter was used as shRNA-producing lentiviral vector (derived from pHR´SINcPPT-SEW [35] by exchange of GFP-WPRE cassette by the RFP gene).

### Lentivirus generation

HEK293T cells were transfected with 50 µg of either targeting construct, 12.5 µg pMD.G VSV-G (encoding the vesicular stomatitis virus envelope glycoprotein) and 50 µg pCMVΔR8.2 (packaging plasmid) by using the calcium phosphate technique. 24 h after transfection, spent medium was replaced by fresh medium containing 10 mM sodium butyrate. 48 and 72 h after transfection, supernatants of each preparation were harvested, pooled, chromatographically purified and concentrated with Lenti-X™ Maxi Purification Kit (TaKaRa) according to the instructions of the manufacturer before freezing in aliquots at − 70 °C.

### Lentiviral infection of MSCs

mMSCs were seeded at 5,000 cells/cm^2^ in T25 flasks the day before infection. The viral infection mix (2 ml/ T25) consisted of MSC medium, the respective virus (5 * 10^5^ viral particles per cell) and 8 µg/ml polybrene (Sigma S-2667). For infection, the medium was removed completely and replaced by the infection mix. Culture vessels were shaken for 30 min at room temperature, 2 ml MSC medium were added and the cells were allowed to recover overnight in the incubator. The infection was repeated as described after 24 h. 24 h after the second infection the virus mix was replaced by 5 ml MSC medium. Successful infection was verified by expression of RFP fluorescence. The cells were further incubated. At the earliest on day 8 after the second infection the cells were used for the experiments. Flow cytometric analysis for cell surface antigens and RFP expression was performed on day 16 after infection under S1 conditions.

### LPS-induced signalling

Native or lentivirally modified mMSCs were grown until 80% confluence in T25 flasks. For analysis of kinase activation, medium was changed to FGF2-free conditions (DMEM with 2 mM glutamine, 10% FCS, 1% 100 × penicillin/ streptomycin) for 1 h. Cells were then stimulated with 1 µg/ml LPS or left untreated (addition of medium for control reactions) for the time periods indicated in the figures (5 to 360 min). Subsequently, cells were directly lysed in 1% (w/v) Nonidet P-40, 150 mM NaCl, 20 mM Tris, pH 7.5, 2 mM EDTA, 50 mM NaF, 1 mM Na_4_P_2_O_7_, supplemented with protease inhibitors (Protease Inhibitor Tablets, Roche Applied Science, Mannheim, Germany) and stored at —80 °C until analysis by Western blotting for activation of kinases. For analysis of gene induction, medium was changed to FGF2-free conditions in the presence of 10% FCS overnight. The following day, mMSCs were exposed to a final concentration of 5 µM TAK1 inhibitor 5Z-7-oxozeanol (concentration according to [36]) or an identical volume of the solvent dimethylsulfoxide for exactly one hour before addition of 1 µg/ml LPS for 1, 2, 3 and 5 days. 24 and 48 h after addition of LPS, another 5 µM 5Z-7-oxozeanol was added. At the end of the respective incubation times medium was removed, and cells were washed with PBS and lysed with TRI Reagent (Ambion/ Thermo Fisher Scientific) for RNA isolation and subsequent gene expression analysis.

### Western blotting

Total protein was analysed by reducing sodium dodecyl sulphate polyacrylamide gel electrophoresis and subsequent Western blotting. Primary antibodies were as follows: TAK1 (D94D7 #5206), TAK1 T184p (#4537), TAK1 T187p (#4536), TAK1 T184p T187p (#4531), JNK (#9252), p-JNK (#9251), p38 (#9212), p-p38 (#9211), IκBα (#9242), IκBα S32p (#2859; all from Cell Signalling), all diluted 1: 1000. TAK1 S439p/S412p was from Invitrogen (PA5-39,743), also diluted 1: 1000. The secondary antibody goat anti-rabbit IgG (H + L), horseradish peroxidase-conjugated (Dianova) was used at a dilution of 1:10,000. For detection, Radiance Q reagent (Azure Biosystems, Dublin, USA) was used in conjunction with the Azure c600 system for detection of chemiluminescence (Azure Biosystems, Dublin, USA). Densitometric analysis of blots was performed with ImageJ 1.53c following the published protocol [37].

### RNA isolation and cDNA synthesis

Cell lysates in 1 ml TRIzol reagent (Ambion) were mixed with 100 µl 1-bromo-3-chloropropane (Merck) and centrifuged for 15 min at 20,000 × g and 4 °C. The upper layer was carefully collected, treated with 500 µL 2-propanol for 5 min and centrifuged for 30 min at 20,000 × g and 4 °C. The RNA pellet was washed with ice-cold 80% ethanol, dried and subsequently dissolved in 20 µL of deionized water. RNA concentration was calculated by absorption at 260/280 nm. Synthesis of cDNA with MMLV reverse transcriptase was performed with 1 µg of RNA and using oligo-dT_18_ primers according to the manufacturer´s protocol (Invitrogen).

### Semi-quantitative RT-PCR

Semi-quantitative RT-PCR was performed using GoTaq DNA polymerase according to the instructions of the manufacturer (Promega). The thermocycler conditions consisted of 90 s at 94 °C followed by a maximum of 35 cycles of 20 s at 94 °C, 20 s at the respective annealing temperature, 30 s at 72 °C, and 15 min at 72 °C for the final extension. All reactions were performed in the linear range by appropriate choice of cycling conditions. HPRT was used as housekeeping gene for normalization. PCR products were analysed by electrophoresis in 1 or 2% (w/v) agarose gels with 0.5 µg/mL ethidium bromide. A 100 bp-marker (Fermentas) was used for size estimation. DNA bands were densitometrically analysed with ImageJ 1.44p (National Institutes of Health). Primer sequences, length of amplification products and annealing temperatures were as follows: HPRT fwd.: 5´-TCAACGGGGGACATAAAA, HPRT rev.: 5´-ATTCAACTTGCGCTCATCTT, 348 bp, 51 °C; IL-6 fwd.: 5´-GATGCTACCAAACTGGATATAATC, IL-6 rev.: 5´-GGTCCTTAGCCACTCCTTCTGTG, 269 or 210 bp (depending on transcript variant), 57.4 °C; TAK1 fwd.: 5´-CAACTCAGCCACCAGCACAGG, TAK1 rev.: 5´-GACTGCGAGCTGGCTTCTCTG, 505 or 424 bp (depending on isoform), 60.0 °C.

#### Quantitative real-time PCR analysis for TAK1

Quantitative real-time PCR analyses were performed using the Applied Biosystems® StepOnePlus instrument (Life Technologies). Each sample was measured in duplicate and the mean values were calculated. The gene-specific assays as well as the Fast Advanced Mastermix were purchased from Life Technologies: *Rps29* Mm02342448_gH (housekeeping gene), *Tak1 (Map3k7)* Mm00554514_m1. Analyses were implemented according to the manufacturer´s instructions. Data were evaluated by the delta C_T_ method with ∆C_T_ = C_T_ (cycle threshold, gene of interest) minus C_T_ (cycle threshold, housekeeper, here *RPS29*), resulting in relative gene expressions of 2^−∆CT^.

### Transwell assays

mMSCs (native or lentivirally modified) were seeded into T25 flasks (125,000 cells/flask). Cells were stimulated with 1 µg/ml LPS or left untreated overnight. If applicable, a goat polyclonal IL-6 antibody (R&D systems AF-406-NA) or a normal goat IgG control antibody (R&D systems AB-108-C) were added (final concentration: 2 µg/ml). Triplicates (600 µl each) of the cell culture supernatants (filtrated, 0.45 µm) were transferred into a 24-well plate. Transwell inserts (polyethylene terephthalate, pore diameter 3 µm Corning #3472) were used. 125,000 splenocytes in 100 µl splenocyte medium were added into the inserts. 3 h later the number of splenocytes that had migrated into the medium containing lower chambers was determined by a Neubauer chamber.

### Mouse cytokine and angiogenesis array

mMSCs (native or lentivirally modified) were seeded into T25 flasks and cultured in starvation medium (88% DMEM with 1 g/l glucose: Biochrom F 0415, 5% foetal calf serum, 2 mM glutamine solution, 1 ng/ml FGF2, 1% 100 × penicillin/ streptomycin mixture) in the presence or absence of LPS (1 µg/ml) for 72 h. The supernatants were subsequently analysed according to the manufacturer´s instructions. The mouse cytokine array panel A #ARY006 (40 analytes) and angiogenesis array ARY015 (53 analytes), both from R&D Systems, were used.

### Statistics

The normal distribution of the data was tested with the Shapiro–Wilk test. The statistical multi-group comparisons were performed using a one-way ANOVA analysis and a post hoc Tukey test (within GraphPad Prism8). For the two-group comparisons a paired or unpaired (depending on the experimental set-up) Student’s *t* test were used. If necessary, the p-values were Bonferroni corrected.

## Results

### mMSC isolation and characterization

mMSCs were isolated from the tibia and femur of C57BL/6 mice. They exhibited plastic-adherent growth and a spindle-shaped fibroblastoid morphology typical of MSCs (Suppl. Figure S1A). The expression of representative cell surface antigens was checked by flow cytometry and corresponded to the expectations for murine MSCs in that CD44 and Sca-1 were present, while CD45 and CD90 were almost absent and CD34, CD73 and CD105 were present partially (Suppl. Figure S1B, C). In vitro differentiation into the three mesenchymal cell lineages (adipocytes, osteoblasts and chondrocytes) was confirmed by histological staining with Oil Red O, for Alkaline Phosphatase activity or with Alcian Blue and Safranin Orange and supported the MSC nature of the cell population (Suppl. Figure S1D).

### LPS-mediated signalling in mMSCs

**Fig. 1:**
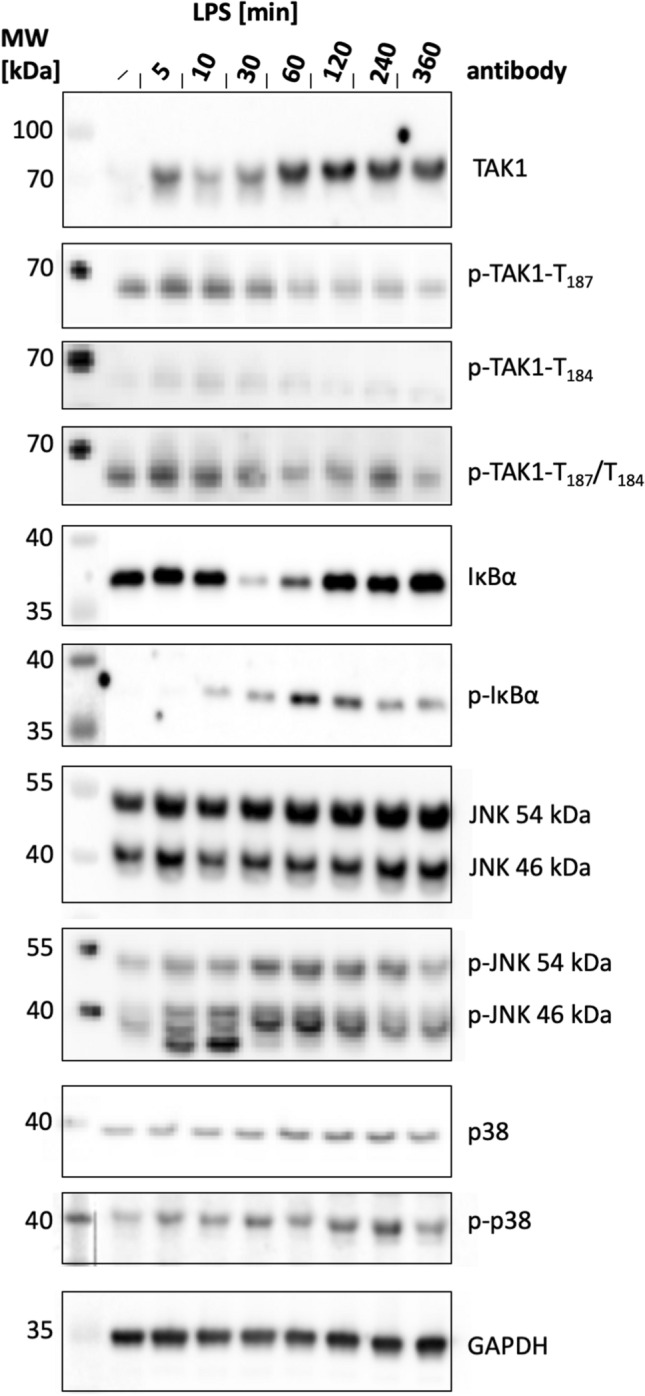
Stimulation of native murine MSCs with 1 µg/ml LPS for different time points. Western blot analyses for protein kinases involved in LPS signal transduction were performed as detailed in Materials and methods. Note that the molecular weight of the bands detected with the different phospho-TAK1 antibodies is lower than the molecular weight of total TAK1, probably arising from non-specific staining

Native MSCs were seeded into culture vessels, allowed to attach and proliferate until 80% confluence, then conditioned in MSC growth medium without FGF2 for 1 h, and subsequently stimulated with 1 µg/ml LPS for different time periods between 5 and 360 min. For these experiments, an ultrapure formulation of LPS without contaminating lipoproteins was used which, according to the manufacturer, only activates TLR4 but not TLR2 in contrast to less pure LPS preparations. The phosphorylation of different mitogen-activated protein kinases, including TAK1, was assessed as an indication of LPS-dependent TLR4 activation (Fig. 1). Interestingly, the LPS addition led to a substantial onset of TAK1 synthesis (isoform TAK1a only) in MSCs after one hour (Fig. 1). In contrast, phosphorylation of TAK1 was a relatively fast event and occurred already 5 min after LPS addition at threonine 187 (T187) as revealed by an antibody specific for TAK1 T187. This phosphorylation was transient and was reduced 30 min after LPS addition (Fig. 1). This phosphorylation of T187 was also confirmed by a double-specific antibody for T187/T184, while for TAK1 T184 low phosphorylation levels only were monitored (Fig. 1). Some basal phosphorylation seemed to be present in the absence of LPS stimulation with both antibodies. All three antibodies – the single phospho-specific as well as the double phospho-specific ones – have repeatedly been used in the literature [38–40]. This issue will be followed up in the discussion section. LPS-dependent phosphorylation of serine 192 (S192) or S412 was not observed in mMSCs (data not shown).

TAK1 downstream signalling mediators were LPS dependently activated to a different extent. The activation of NFκB prominently involved IκBα as a signalling mediator. Previous work has established that signal-dependent phosphorylation of NFκB is followed by proteasome-mediated degradation resulting in the release and nuclear translocation of active NFκB. In the present study, substantial IκBα phosphorylation was observed starting 10 min after stimulation concomitant with the expected degradation of the IκBα protein moiety which was most pronounced after 30 min of stimulation (Fig. 1). LPS application also led to phosphorylation of both JNK isoforms, p46 and p54, peaking 10 to 30 min after LPS stimulation. In mMSCs only very moderate LPS-dependent phosphorylation levels were detected for mitogen-activated protein kinase p38 (Fig. 1). On the basis of these observations we selected a 30-min period for subsequent LPS stimulation experiments with genetically modified mMSCs.

### TAK1 knockdown in mMSCs

**Fig. 2:**
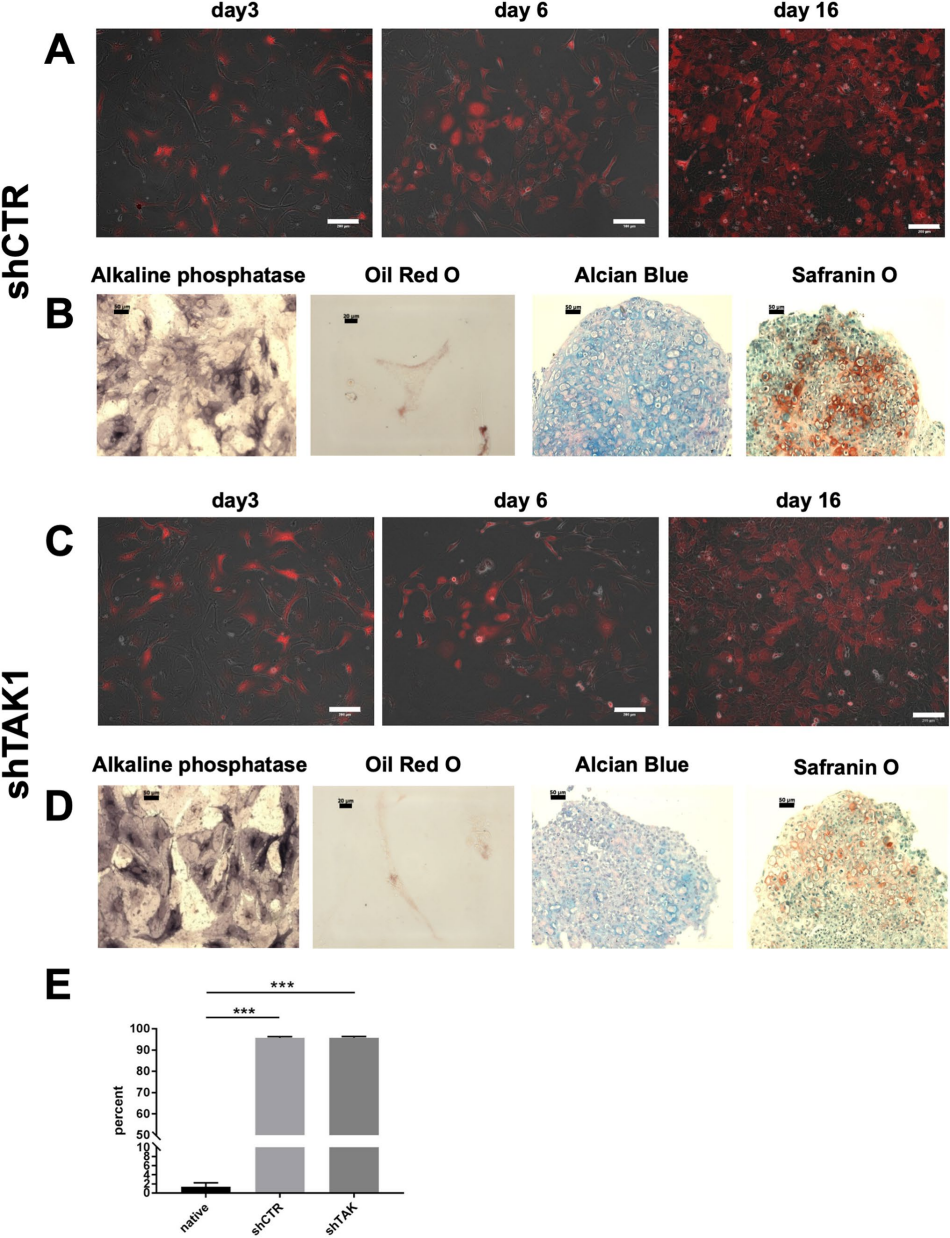
Lentiviral modification of murine MSCs: morphology, infection efficiency and differentiation. A, B: Infection with control lentivirus shCTR and C, D: Infection with lentivirus shTAK1. A, C: overlay of RFP fluorescence and phase contrast microscopy. Scale bars: 200 µm. B, D: In vitro differentiation and histocytochemical staining of mMSCs (passage 8). Osteogenic differentiation was assessed by alkaline phosphatase staining at day 7 post-confluence. Scale bar: 50 µm. Adipogenic differentiation was evaluated with Oil Red O staining at day 21 post-confluence. Scale bar: 20 µm. Chondrogenic differentiation was detected by staining with Alcian Blue stain and Safranin O at day 21 post-confluence. Scale bars: 50 µm each. E: Quantification of RFP fluorescence from flow cytometry, four independent experiments. Dead cells were excluded from analysis. For the pairwise comparisons the Students t-test was used ***:p < 0.001

**Fig. 3:**
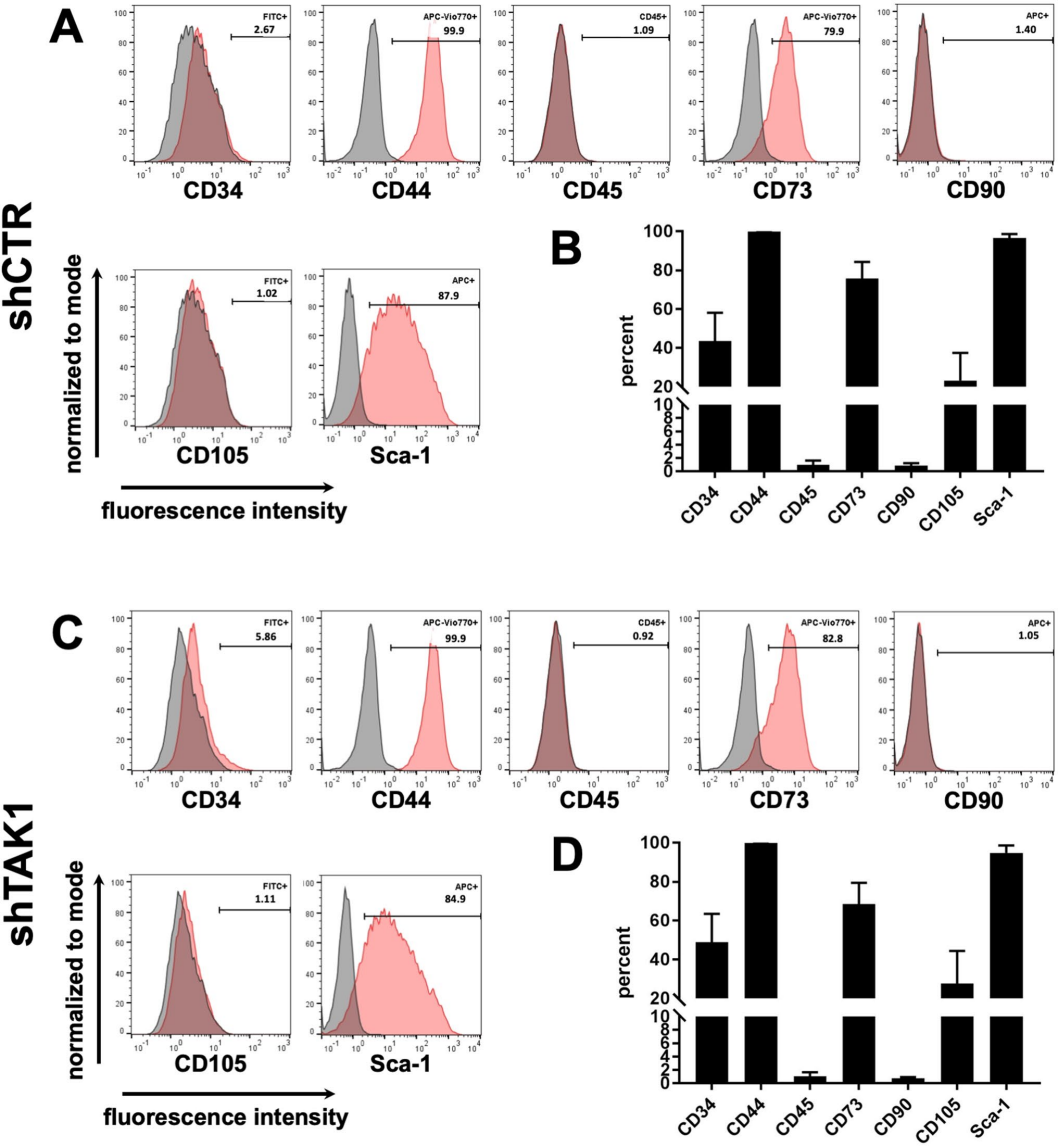
Lentiviral modification of murine MSCs: cell surface antigens. **A, B**: Infection with control lentivirus shCTR and **C, D**: Infection with lentivirus shTAK1. **A, C**: Exemplary flow cytometric profiles of mMSCs infected with lentivirus from one out of four independent experiments, 16 days after the second transduction. **B, D**: Flow cytometric data (mean ± SEM) for the four independent experiments

The TAK1 expression in mMSCs was modulated by shRNA-mediated downregulation. One shRNA targeting TAK1 (shTAK1) that has successfully been used in previous studies (see methods section) was lentivirally expressed under the control of a H1 promoter and compared with a non-targeting control virus containing a random sequence (shCTR). In addition to shRNAs, these lentiviruses directed the expression of red fluorescent protein (RFP) from an SFFV promoter. Figure 2A, C documents the cell morphology by immunofluorescence three, six and 16 days after lentiviral infection, with the later time point demonstrating the stability of the genetic modification. Quantification by flow cytometric analyses (more sensitive than analysis by microscopy) of RFP fluorescence 16 days after transduction indicated an overall infection efficiency of over 90% (Fig. 2E). Due to this high infection efficiency the cells were directly used for all subsequent experiments. Prominent cell surface expressions of CD44, CD73 and Sca-1 were documented as in native MSCs (Fig. 3; data for native MSCs in Suppl. Fig. S1B, C), while CD45 and CD90 remained negative as observed in native mMSCs. The surface expressions of CD34 (known to be expressed on murine MSCs) [41] and CD105 were reduced after lentiviral modification (not statistically significant). Despite these alterations, in principle the selected surface antigen expressions were stable. The influence of TAK1 expression on mesenchymal differentiation was assessed by in vitro differentiation of lentivirally modified cells into the osteogenic, chondrogenic and adipogenic lineage for up to 21 days and subsequent analysis by cytochemical staining. Formation of adipocytes and osteoblasts were not substantially affected by TAK1 downregulation. Interestingly, however, a notably lower number of chondrocytes seemed to have formed in MSCs with lentivirally downregulated TAK1 (Fig. 2D compared to Fig. 2B) although this issue was not further followed up.

### TAK1 downregulation interferes with LPS-mediated signalling in mMSCs

**Fig. 4:**
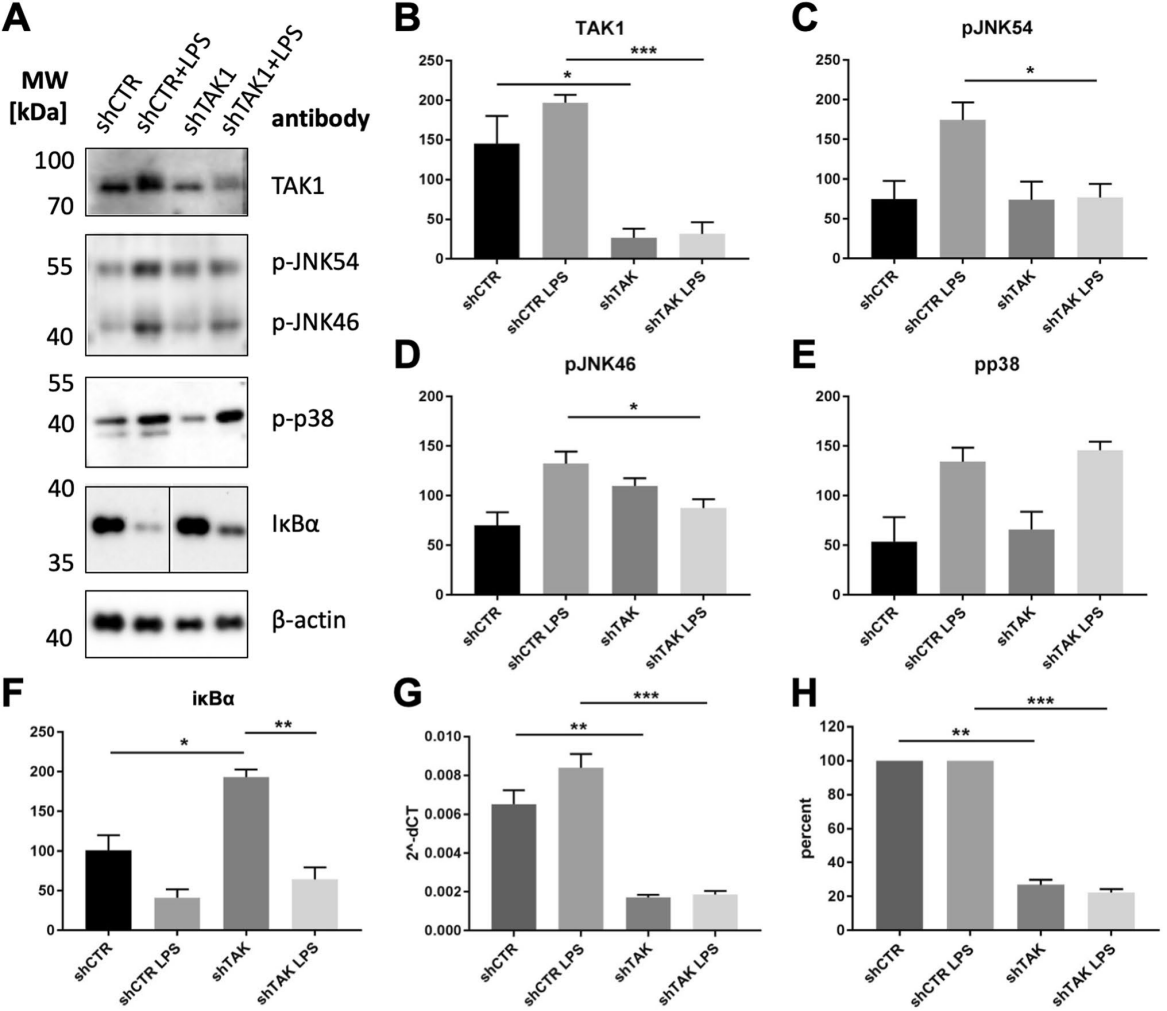
TAK1 downregulation by lentiviral-mediated shTAK1 expression in mMSCs affects LPS-stimulated signalling pathways. After lentiviral modulation of TAK1 expression inflammatory signalling pathways were assessed after LPS stimulation (LPS: 1 µg/ml, 30 min). **(A)** p38, IκB and JNK pathways were analysed after Western blotting with antibodies specific for activated signalling factors. One representative result of three individual experiments is shown. **(B-F)** The graphs demonstrate densitometric analysis of TAK1 **(B)** and JNK **(C and D)**, p-p38 **(E)** and IκBα pathways **(F)**, mean ± SEM. The average density level of each band from three individual experiments was corrected for actin loading controls. Densitometric analysis of blots was performed with ImageJ 1.53c following the published protocol [37]. For the pairwise comparisons the Students t-test was used *: p < 0.05, **: p < 0.01, ***: p < 0.001. **(G)** Quantification of TAK1 expression by quantitative real-time PCR in the four experimental groups, three independent experiments. Relative gene expression analysis (2^−ΔCt^), mean ± SEM. **(H)** The data from **(C)** were set to 100% and the downregulation of TAK1 expression was calculated for the non-stimulated samples and the LPS-stimulated samples, respectively

After targeting with the shTAK1 virus, TAK1 protein levels were efficiently downregulated as demonstrated by Western blotting and quantitative real-time PCR (Fig. 4). The TAK1 protein level was reduced to 18% (non-stimulated samples) or 16% (LPS-stimulated samples) of the control TAK1 level (Fig. 4A, B). Quantitative real-time PCR detected 26.8% (non-stimulated samples) or 22.3% (LPS-stimulated samples) of the control TAK1 level (Fig. 4G, H).

shTAK1-dependent downregulation of TAK1 in mMSCs led to stabilization of IκBα prior to and after LPS induction although statistical significance was achieved only in the absence of LPS (Fig. 4F). This finding confirms that TAK1 is involved in signal propagation of LPS via TLR4 in mMSCs as previously described for fibroblasts [42].

Lower TAK1 levels by virally expressed shTAK1 led to significantly reduced phospho-c-Jun N-terminal kinase (JNK: p46 and p54 isoforms) phosphorylation levels in mMSCs (Fig. 4C and D). This finding confirms that TAK1 is involved in signal propagation of LPS via JNK as previously observed for monocytes [30]. In contrast, downregulated TAK1 in mMSCs did not affect p38 phosphorylation levels indicative for a lack of TAK1 participation in LPS-mediated p38 activation in this particular cell type (Fig. 4E).

### TAK1 downregulation interferes with lymphocyte recruitment of LPS-activated MSCs

**Fig. 5:**
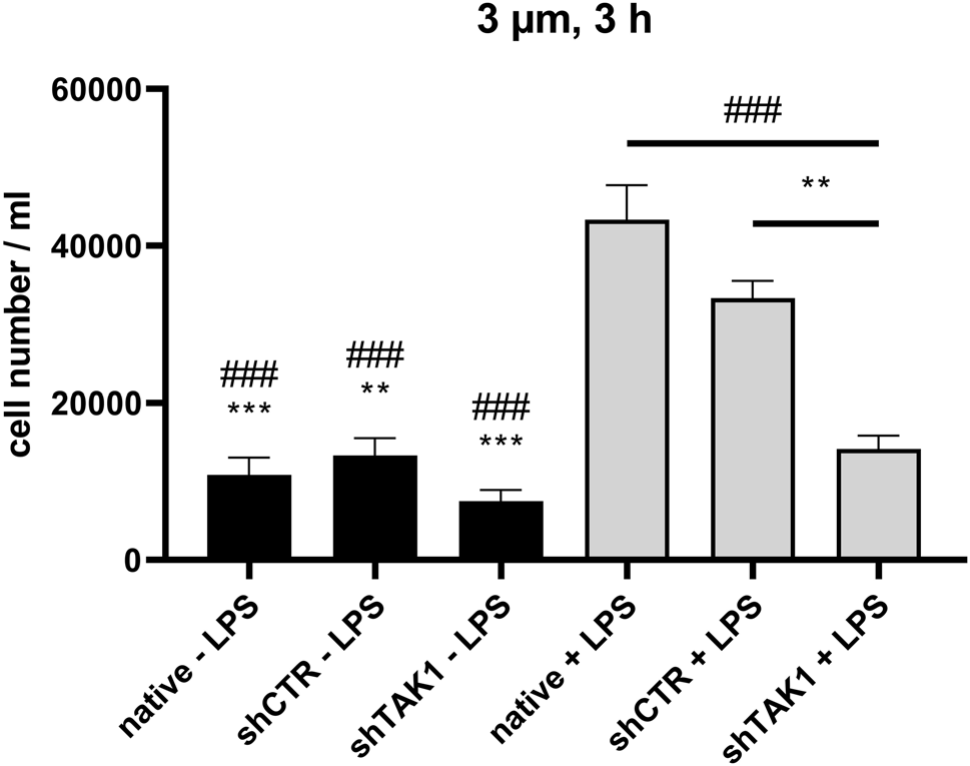
Lymphocyte recruitment by TAK1 genetically modified mMSCs. The experiment was conducted in triplicates. The Data are displayed as mean ± SEM. Statistical analysis was performed by one-way ANOVA analysis and a post hoc Tukey test. The significance of the statistics is given in comparison to native + LPS (### p ≤ 0.001) and shCTR + LPS (** p ≤ 0.01 and *** p ≤ 0.001). All other combinations were not significant in the one-way ANOVA and the following Tukey test

We next addressed the biological relevance of TAK1-mediated signalling in regard to the ability of LPS-treated mMSCs to recruit murine splenocytes (composed of about 80% lymphocytes). Conditioned medium from mMSCs that had been stimulated overnight with LPS was assessed as potential chemotactic stimulus on the migration of freshly isolated splenocytes (Fig. 5). Medium from LPS-stimulated mMSCs (native and shCTR) favoured splenocyte migration. Figure 5 shows that during 3 h exposure of splenocytes to conditioned medium, medium from LPS-activated native mMSCs induced the highest migration with 43,333 cells/ml (95% confidence interval (CI) [37786, 48800]). The migration of splenocytes exposed to medium from LPS-stimulated shCTR-mMSCs was significantly higher than after exposure to medium from shTAK1 mMSCs ((33,333 ± 3118 cells/ml (95% CI [27786, 38800]) versus 14,167 cells/ml (95% CI [8620, 19714], p ≤ 0.01)). In the absence of LPS, migration of splenocytes after exposure to medium from shTAK1 mMSCs (7500 cells/ml (95% CI [1953, 13047]) was not significantly reduced in comparison to medium from shCTR-mMSCs (13,333 ± 3118 cells/ml95% CI [7786, 18800]). In conclusion, TAK1 signalling affected the capacity of LPS-treated mMSCs to recruit lymphocytes.

### TAK1 regulates IL-6 expression in LPS-activated mMSCs

To pinpoint cytokines which were secreted from MSCs after LPS stimulation in a TAK1-dependent fashion, we used commercially available cytokine arrays from R&D Systems, the “cytokine panel A” array allowing the profiling of 40 analytes and the “angiogenesis” array with 53 analytes. 9 analytes were identical on both arrays so that a total of 84 different factors were assessed. mMSC populations were stimulated with LPS for 72 h in medium with a reduced (5%) amount of foetal bovine serum. Native mMSCs were left untreated for comparison. The cell-conditioned medium was recovered and analysed on the “cytokine” and “angiogenesis” arrays.

**Fig. 6:**
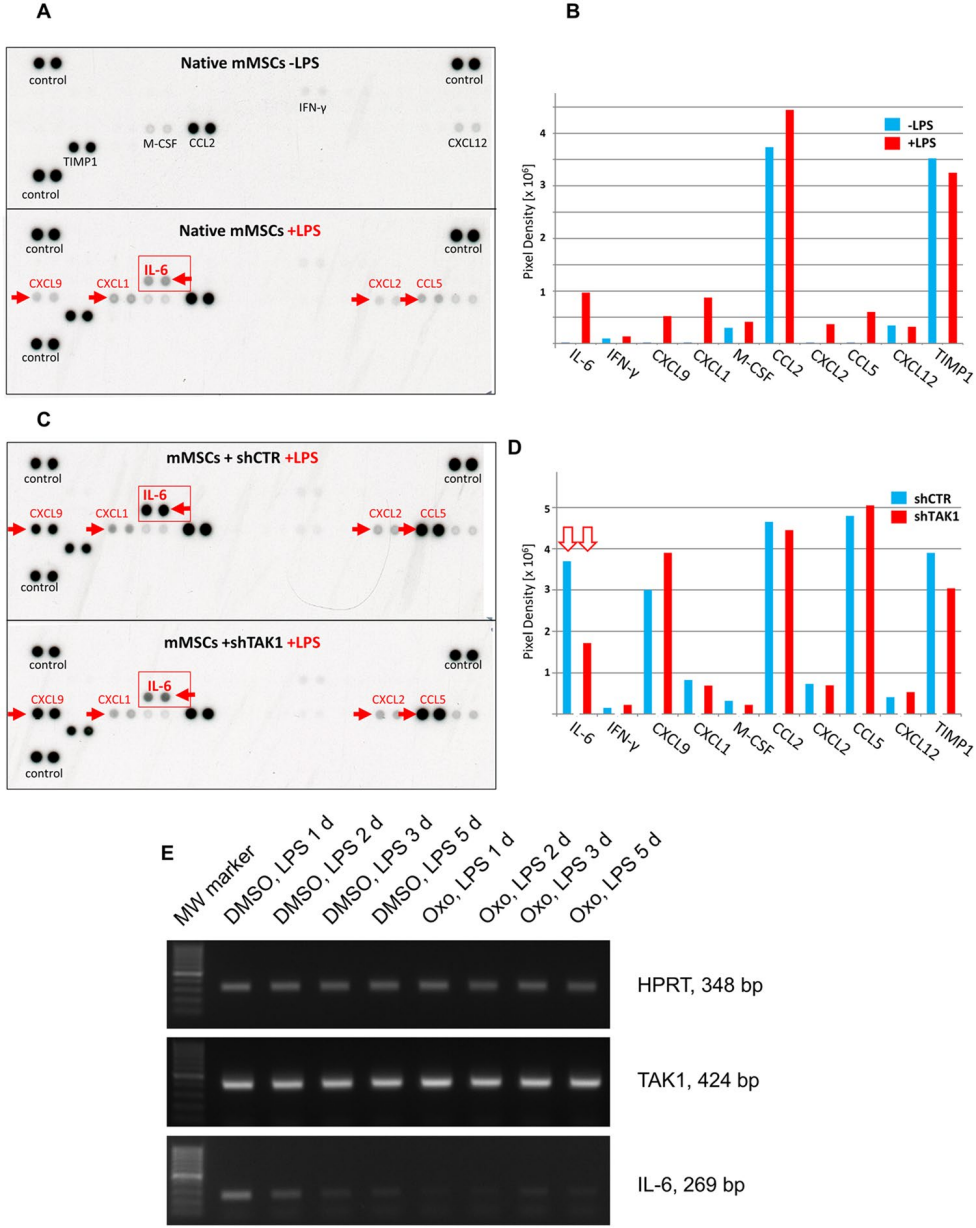
Analysis of LPS‐secreted factors in murine MSCs in a TAK1‐dependent mode. (A) LPS‐stimulated cytokine secretion in native mMSCs. mMSCs were cultivated in reduced serum levels (5%) for 72 h in the presence or absence of LPS (1 μg/ml). LPS induces/upregulates 5 factors (red arrows) out of 10 secreted cytokines in native mMSCs. **(B)** Expression levels were quantified as pixel density as determined by image analysis. **(C)** LPS‐stimulated cytokine secretion in mMSCs infected with lentiviruses encoding small hairpin control RNA (shCTR) or shRNA specific for TAK1 (shTAK1). IL‐6 is the only secreted cytokine detected which is substantially regulated by TAK1 signalling. **(D)** Expression levels were quantified as pixel density as determined by image analysis. TAK1 downregulation leads to a reduction of the IL‐6 secretion level to 46.5% (red arrows). **(E)** Native mMSCs were exposed to 5 µM TAK1 inhibitor 5Z-7-oxozeanol or solvent one hour prior to stimulation and then stimulated with LPS (1 µg/ml) for the time periods indicated. MW marker 100 bp (range 100—1000 bp)

Five factors were detected as secreted by native MSCs on the cytokine array (Fig. 6A). In addition to these, IL-6, CXCL1, -2, -9 and CCL5 were induced by LPS (Fig. 6B). To monitor as to whether or not these factors were LPS upregulated by TAK1-mediated signalling, the supernatant of LPS-stimulated MSCs infected with either control virus (shCTR) or with virus specific for TAK1 (shTAK1) was collected and investigated as before. Lentivirally infected and LPS-stimulated MSCs secreted the identical factors as native LPS-stimulated cells, interestingly though the secretion level of several factors (e.g. CXCL9, CCL5, IL-6) was strongly enhanced by the lentiviral infection as such, indicating the activation of alternative/additional signalling pathways by the lentiviral system (Fig. 6C, D). Surprisingly, the only secreted factor that was severely affected by TAK1 downregulation was IL-6 (Fig. 6C, D).

Analysis of the angiogenesis array revealed that LPS induced/upregulated 7 factors (red arrows) out of 21 secreted after LPS treatment of mMSCs (Suppl. Figure S2). The combined profiling data with the two arrays showed that mMSCs secreted 26 factors in total, 10 of these were LPS induced/upregulated (Suppl. Figure S3); however, as already stated, IL-6 was the only LPS-induced/upregulated and secreted factor exhibiting a substantial TAK1-dependent regulation. Based on the pixel density, lentiviral-mediated downregulation of TAK1 reduced the level of IL-6 expression in LPS-treated cells to 46.5% (Fig. 6D).

To confirm the TAK1-dependent mode of IL-6 induction after LPS stimulation and to exclude that the observed TAK1 dependence effect might be caused by the lentiviral infection procedure itself or by increased protein turnover, we investigated IL-6 mRNA levels in LPS-stimulated mMSCs. We found that IL-6 mRNA was induced by 1 µg/ml LPS within 24 h and was sensitive to TAK1 signalling, indeed, since the low-molecular weight TAK1 inhibitor 5Z-7-oxozeanol efficiently interfered with LPS-dependent IL-6 transcription (Fig. 6E), substantiating our lentiviral analyses. No induction of IL-1 or TNF-α gene expressions were detected, in accordance with the absence of detection by the cytokine arrays.

In conclusion, lentiviral infection and LPS stimulation of mMSCs both change the secretory profile of MSCs. Among the 10 LPS-induced/upregulated factors IL-6 was the only cytokine that was secreted in a TAK1-dependent way as evidenced by the arrays. In light of the important role of TAK1 in inflammatory pathways, this low number of secreted factors in a TAK1-dependent mode may seem surprising. Moreover, no LPS induction of TNF-alpha or IL-1β-mRNA synthesis was detected at any time point investigated (data not shown). We will refer to these facts in the discussion section.

### Lymphocyte recruitment by LPS-activated MSCs is mediated by IL-6

**Fig. 7:**
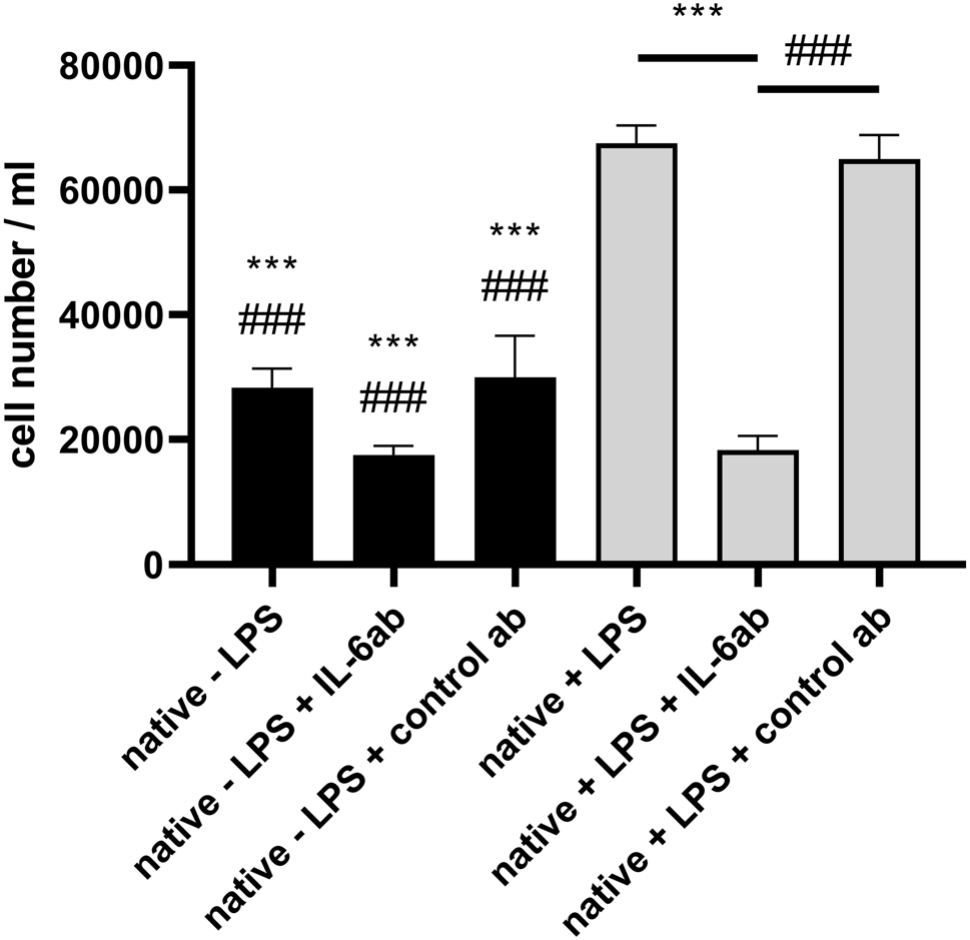
Identification of IL-6 as a secreted factor influencing splenocyte migration. LPS stimulation was performed overnight in the presence of IL-6 antibody or a control antibody. The experiment was conducted in triplicates. The data were evaluated by a one-way ANOVA and a post hoc Tukey test in comparison to native + LPS (*** p ≤ 0.001) and native + LPS + control ab (### p ≤ 0.001). Other combinations were not significant in the one-way ANOVA and the following Tukey test

Above, we showed in transwell experiments that the capacity of LPS-stimulated mMSCs to recruit lymphocytes/splenocytes is TAK1 dependent (Fig. 5). Since IL-6 was the only cytokine which we detected as secreted and modulated by TAK1 signalling from LPS-stimulated mMSCs, we reasoned that IL-6 would be responsible for the observed TAK1 dependence of lymphocyte recruitment. We, therefore, repeated the transwell experiments with splenocytes in the presence or absence of antibodies specifically neutralizing IL-6 activity. As a control a non-relevant antibody was added to the medium (Fig. 7). Medium from native non-stimulated mMSCs allowed a low but distinct splenocyte migration. Addition of IL-6 antibodies to non-stimulated mMSCs supernatant further but not significantly decreased the extent of migration, suggesting a low rate of lymphocyte recruitment mediated by base level of IL-6 which was not detected in the array experiments (Fig. 7). Addition of the control antibody to the supernatant of unstimulated MSCs confirmed this tendency since in this case a similar number of lymphocytes as in the non-treated state were able to migrate (Fig. 7).

In contrast, supernatant of LPS-stimulated MSCs substantially increased splenocyte migration in the absence of neutralizing IL-6 antibodies (Fig. 7: p ≤ 0.001 compared to medium from non-stimulated cells). The presence of the IL-6 neutralizing antibodies significantly reduced the number of migrated cells (about 3.5-fold; Fig. 7: p ≤ 0.001 compared to control antibody). Specifically, the number of migrated cells using conditioned medium from LPS-stimulated mMSCs containing IL-6 antibodies (18300cells/ml; 95% CI [9414, 25586]) was similar to the number of migrated cells from non-stimulated mMSCs containing IL-6 antibodies (17,500 cells/ml; 95% CI [10247, 26419]) (Fig. 7). From this we conclude that in our experimental set-up IL-6 is a major secreted factor responsible for LPS-dependent splenocyte migration, indeed. In summary, we believe to have demonstrated that TAK1 regulates lymphocyte recruitment of LPS-stimulated mMSCs via the cytokine IL-6.

## Discussion

In this study we asked the question in as much the major inflammatory signalling mediator TAK1 might be involved in MSC-dependent lymphocyte recruitment being the prerequisite for MSC-dependent anti-inflammatory and immunosuppressive activities. We reasoned that LPS would be an appropriate way to activate MSCs and to exert these immunosuppressive effects. Fibroblastic cells efficiently respond to LPS in a Toll-like receptor 4 (TLR4)-dependent fashion; moreover, the signalling mediator TAK1 has been pinpointed as the critical pathway for TLR-induced transcription factors nuclear factor κB (NFκB) and activator protein-1 (AP-1) in general [24, 29]. Although, subsequent studies revealed that additional pathways are activated by cytosolic LPS involving caspases and inflammasomes [43, 44]. It has been demonstrated that TAK1 activation requires multiple phosphorylations in its kinase activation loop, such as T184, T187 and S192 [45]. On the basis of results obtained with specific antibodies in mMSCs, efficient LPS-dependent phosphorylation of T187 in the kinase activation loop could apparently be detected with a commercial antibody (Fig. 1). However, interestingly, the molecular weight of all phosphorylated variants is lower than the 70 kDa molecular weight standard band, whereas it is higher for total TAK1 while usually phosphorylation retards electrophoretic mobility. In three independent experiments with TAK1 knockdown, no conclusive data could be obtained with respect to the nature of these bands and whether they are specific to phosphorylated TAK1. In order to share this information with other researchers we decided to display Fig. 1 with inclusion of these data. In response to LPS, the phosphorylation-dependent phosphorylation and downregulation of IκBα were monitored as the prerequisite of an active NFκB transcription factor (Fig. 1). JNK p46/p54 JNK were moderately phosphorylated, while the addition of LPS activated p38 only to a limited extent (Fig. 1).

Administration of lentiviruses encoding shRNAs specific for TAK1 mRNA led to the downregulation to 26.8% (non-stimulated samples) or 22.3% (LPS-stimulated samples) of the control TAK1 level by quantitative real-time PCR (Fig. 4). This downregulation was sufficient to affect the stabilization of IκBα (Fig. 4). The phosphorylation rate of c-Jun-N-terminal kinases also was affected by TAK1 downregulation, while, in contrast, a substantial effect of TAK1 downregulation on p38 phosphorylation was not observed. Possibly, other MAP3K, such as MAP-ERK kinase kinase-1, -2 (MEKK1 and MEKK2), could activate p38 signalling and cause the insensitivity of p38 phosphorylation to TAK1 downregulation [42, 46]. In addition, the above-mentioned inflammasome activation by cytosolic LPS independently of TLR4 ligation could contribute to JNK and p38 activation [43, 44, 47]. We also noticed that variations in knockdown efficiency seemed to be accompanied by variations in the optimal time kinetics for maximum IκBα degradation. Consequently, this delicate system resulted in effects that varied between experiments, with statistical significance only obtained for the pJNK variants and for IκBα in the absence of stimulation.

Here, TAK-1 efficiently regulated splenocyte migration towards the supernatant of LPS-stimulated mMSCs (Fig. 5). Medium from LPS-stimulated mMSCs clearly favoured splenocyte migration in our experimental transwell analyses. Importantly, splenocyte migration was considerably affected by lentiviral downregulation of TAK1, indicating that this inflammatory signalling mediator is involved in the establishment of anti-inflammatory activities of MSCs, indeed. Interestingly, lentiviral infection alone was able to stimulate the LPS-dependent expression of chemokines CCL5 and CXCL9 several-fold, while in native mMSCs these cytokines were only weakly induced by LPS (Fig. 6), indicating that additional signalling pathways are activated by the lentiviral infection procedure. Similarly, increased levels of IL-6 secretion were also observed in lentivirally modified control mMSCs (Fig. 6, shCTR).

The surprising fact was, that, based on the factor profiling, among the 10 LPS-upregulated factors IL-6 was the only factor regulated in a TAK1-dependent fashion (Fig. 6). The cytokine IL-6 possesses the capacity to attract lymphocytes [20]. Here, IL-6 was the most important factor contributing to the LPS-dependent capacity of mMSCs to recruit lymphocytes. The presence of IL-6 neutralizing antibodies reduced the number of migrated cells about 3.5-fold, yet a substantial level of lymphocyte migration was unaffected by the presence of IL-6 neutralizing antibodies (Fig. 7). LPS-induced/upregulated chemokines, like CCL5, CXCL9 and others (Suppl. Figure S3) may, therefore, contribute to the lymphocyte recruitment capacity of LPS-triggered mMSCs, albeit to a lesser extent.

IL-6 is known to direct profound pro-inflammatory and anti-inflammatory effects, reviewed in [48, 49]. Contributing to the inflammatory character of IL-6 is its chemotactic activity for neutrophils, macrophages and T cells [50, 51]. In contrast, IL-6 also possesses potent anti-inflammatory activities [52, 53]. In the latter regard it is highly interesting that LPS-stimulated monocytes interfere with the secretion TNF-α and IL-1 via IL-6 production. The authors suggest that this suppressive effect of IL-6 upon IL- 1 and TNF-α synthesis might also be relevant for fibroblasts [54]. Another study documented that IL-6 ^−/−^ mice possess dramatically elevated levels of IL-1 and TNF-α substantiating an IL-6-dependent interfering influence on the secretion level of these two important pro-inflammatory cytokines [55]. In mMSCs, we did not monitor IL-1 or TNF-α secretion triggered by LPS stimulation (no reaction on the cytokine array B17/18 for IL-1α, B19/20 for IL-1β; E5/E6 for TNF-α), and no gene expression could be documented. A synthesis of cytokines, like IL-1 or TNF-α, in LPS-triggered mMSCs would undoubtedly amplify TAK1 signalling by the activation of their respective receptors resulting in a major inflammatory response.

Therefore, the capacity to activate LPS-mediated TLR4 signalling in mMSCs along the TAK1 pathway seems limited. Activation of the IL-6 signalling machinery may modulate TAK1 signalling by physical and functional interaction of their respective cytoplasmic components. So, interaction of IL-6 activated STAT3 with TAK1 specifically enhances IL-6-induced TAK1-dependent Nemo-like kinase activation, but not TGF-β-induced Nemo-like kinase activation [56]. TAK1 seems to select its specific downstream effector kinases in conjunction with specific scaffold proteins indicating its variability to respond to stimuli differently [56, 57].

LPS-dependent TAK1 activation also depends on the source of the fibroblasts. For example, fibroblasts isolated from the synovium seem even completely unable to mediate TAK1-dependent effects if triggered by LPS [42]. It might be tempting to speculate that IL-6 might be involved in limiting the broad inflammatory impact of TAK1 signalling. In conclusion, TAK1 predominantly mediates efficient expression of only one particular cytokine in LPS-activated mMSCs: IL-6. Secreted from MSCs, IL-6 seems to be able to mediate a plethora of different actions, e.g. from anti-apoptotic [58] to tumour promoting activities [59]. Importantly, IL-6 promotes several crucial anti-inflammatory actions, e.g. in vivo via PGE2 [60] and—as demonstrated in the present in vitro study—that LPS-activated mMSCs use the TAK1 signalling pathway to secrete IL-6 resulting in efficient lymphocytes recruitment as a prerequisite for the downregulation of MSC-mediated inflammatory activities.

## Supplementary Information

Below is the link to the electronic supplementary material.

Supplementary file1 (DOCX 2624 kb)

## Data Availability

The data used to support the findings of this study are included within the article. All data generated or analysed during this study are included in this published article and its supplementary information.
