# Supplementary material for: The inflammatory signalling mediator TAK1 mediates lymphocyte recruitment to lipopolysaccharide-activated murine mesenchymal stem cells through interleukin-6
Source: Mol Cell Biochem. 2021 May 29;476(10):3655–70. doi: 10.1007/s11010-021-04180-8 (PMC8382631; doi:10.1007/s11010-021-04180-8)
Supplement: Supplementary file 1 — Supplementary file1 (DOCX 2624 kb) [file 11010_2021_4180_MOESM1_ESM.docx]

Supplementary Material

**The inflammatory signalling mediator TAK1 mediates lymphocyte recruitment to lipopolysaccharide-activated murine mesenchymal stem cells through interleukin-6**

Beatrice Oelze^1^, Kirsten Elger^2,3^, Patrik Schadzek^2,3^, Laura Burmeister^2,3^, Anika Hamm^2,3^, Sandra Laggies^1^, Virginia Seiffart^1^, Gerhard Gross^1^ & Andrea Hoffmann^1,2,3,&^

^1^ Helmholtz Centre for Infection Research (HZI), Inhoffenstr. 7, 38124 Braunschweig, Germany

^2^ Hannover Medical School, Department of Orthopaedic Surgery, Graded implants and regenerative strategies OE 8893, Stadtfelddamm 34, 30625 Hannover, Germany

^3^ Hannover Medical School, Lower Saxony Centre for Biomedical Engineering, Implant Research and Development (NIFE), Stadtfelddamm 34, 30625 Hannover, Germany

& Corresponding author: Hoffmann.Andrea@mh-hannover.de

**
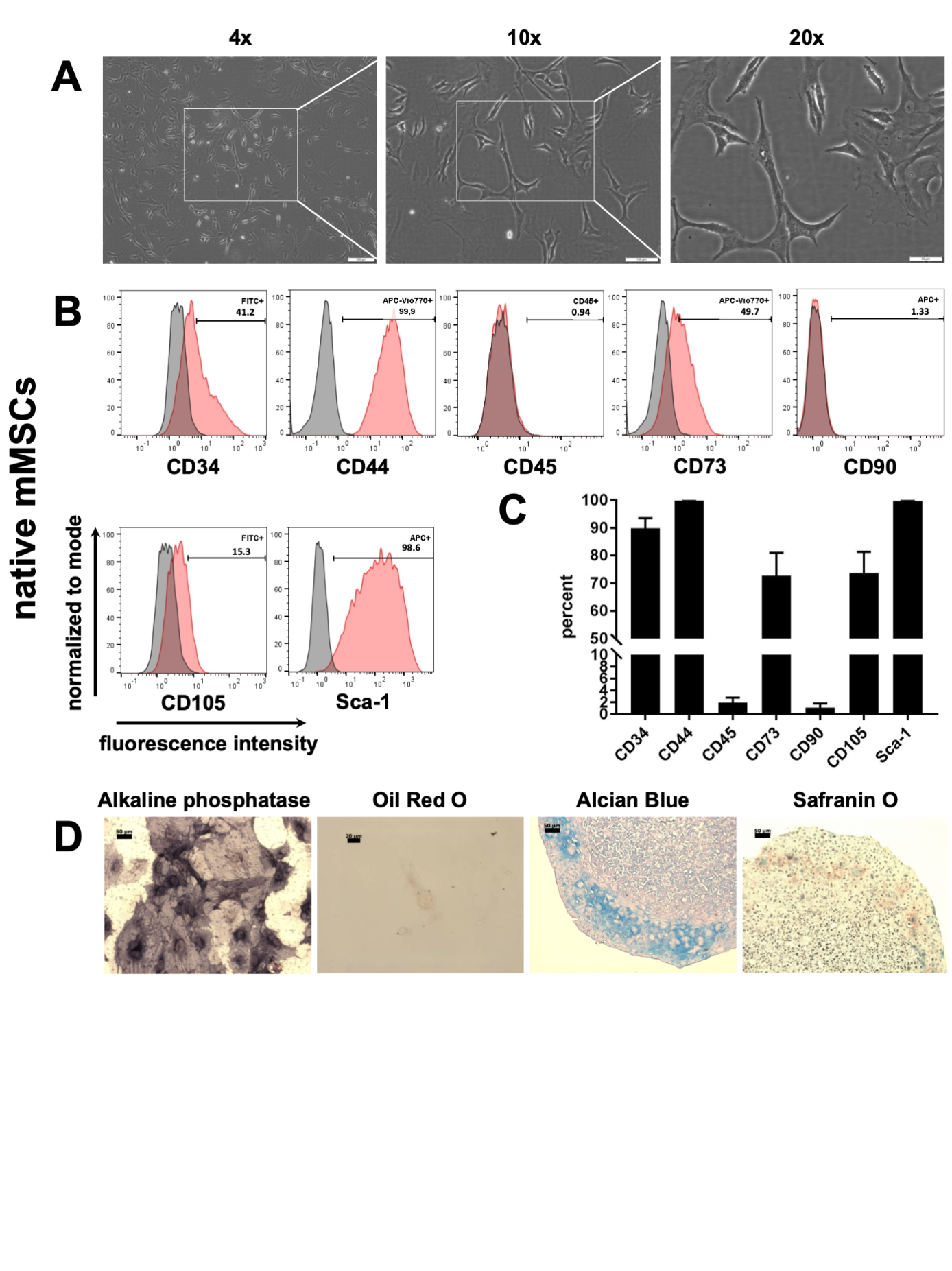
**

**Supplementary Fig. S1. Characterization of native murine MSCs from bone marrow of C57BL/6 mice by morphology, flow cytometry and *in vitro*-differentiation.**

**(A)** Morphology of mMSCs. The cells exhibit an elongated spindle-shaped morphology with large nucleus that is typical of MSCs, passage 11. Scale bars: 200 µm (A), 100 µm (B), 50 µm (C). **(B)** Exemplary flow cytometric profiles of native mMSCs from one out of four independent experiments. Dead cells were excluded from analysis. **(C)** Flow cytometric data (mean ± SEM) for the four independent experiments. **(D)** In vitro differentiation and histocytochemical staining of mMSCs (passage 8) was performed as outlined in the Methods-Section. Osteogenic differentiation was assessed by alkaline phosphatase staining at day 7 post-confluence. Scale bar: 50 µm. Adipogenic differentiation was evaluated with Oil Red O staining at day 21 post-confluence. Scale bar: 20 µm. Chondrogenic differentiation was detected by staining with Alcian Blue stain and Safranin O at day 21 post-confluence. Scale bars: 50 µm each.

**
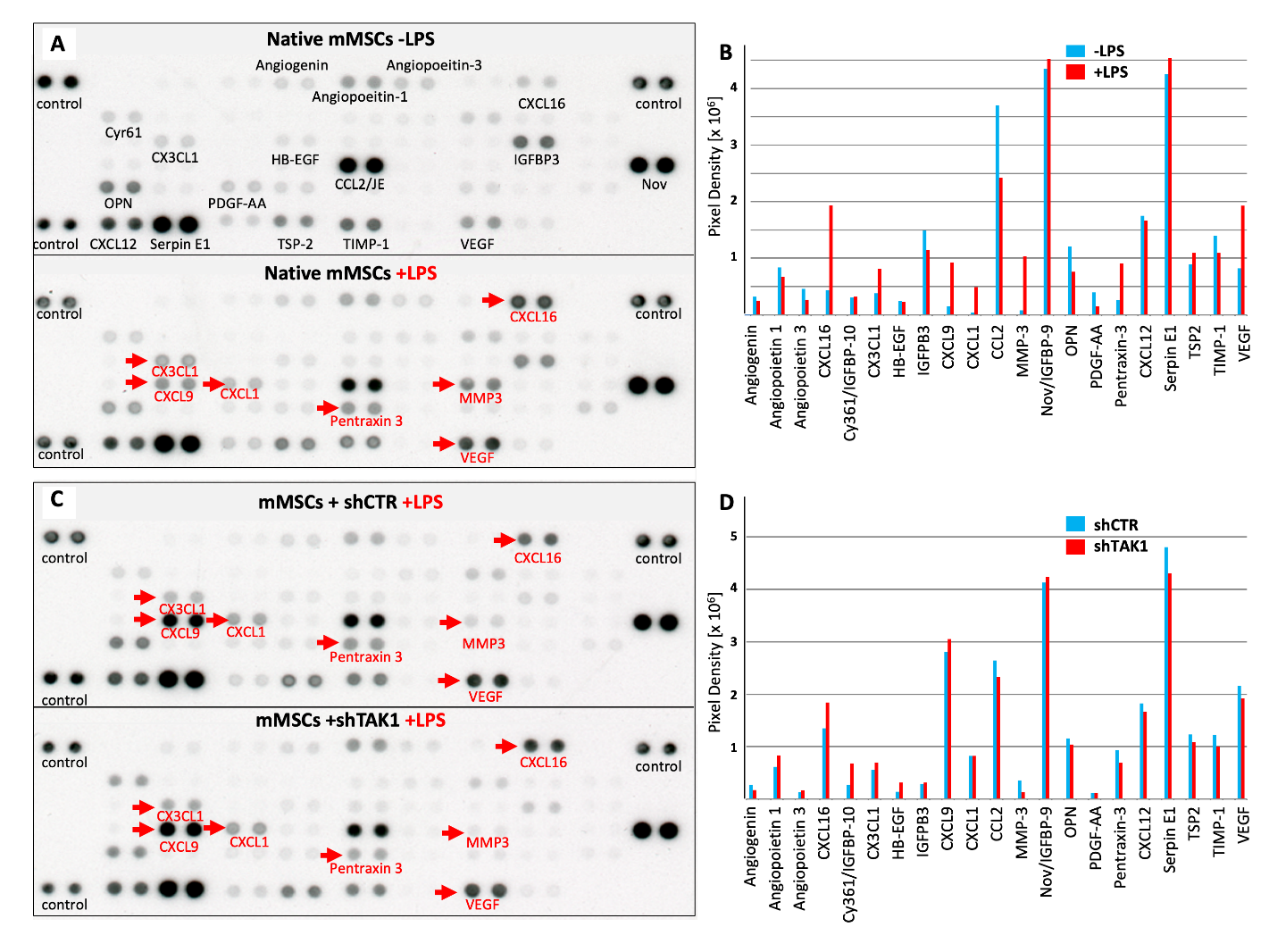
Suppl. Fig. S2: Analysis of LPS‐secreted factors in murineMSCs in a TAK1‐dependent mode in murine MSCs with an Angiogenesis Array (R&D). (A)** LPS‐stimulated cytokine secretion in native mMSCS. mMSCs were cultivated in reduced serum levels (5%) for 72 h in the presence or absence of LPS (1 μg/ml). The supernatant was applied to the Angiogenesis Array from R&D systems as described in the Methods Section. LPS induces/upregulates 7 factors (red arrows) out of 21 secreted cytokines in native mMSCs detected. **(B)** Expression levels were quantified as pixel‐density as determined by image analysis. **(C)** LPS‐stimulated cytokine secretion in mMSCs infected with lentiviruses encoding small hairpin control RNA (shCTR) or shRNA specific for TAK1 (shTAK1). The same 7 factors as in native LPS-stimuated mMSCs are upregulated. No LPS-induced/upregulated secreted cytokine was monitored substantially dependent on TAK1 expression levels. **(D)** Expression levels were quantified as pixel‐density as determined by image analysis.

**
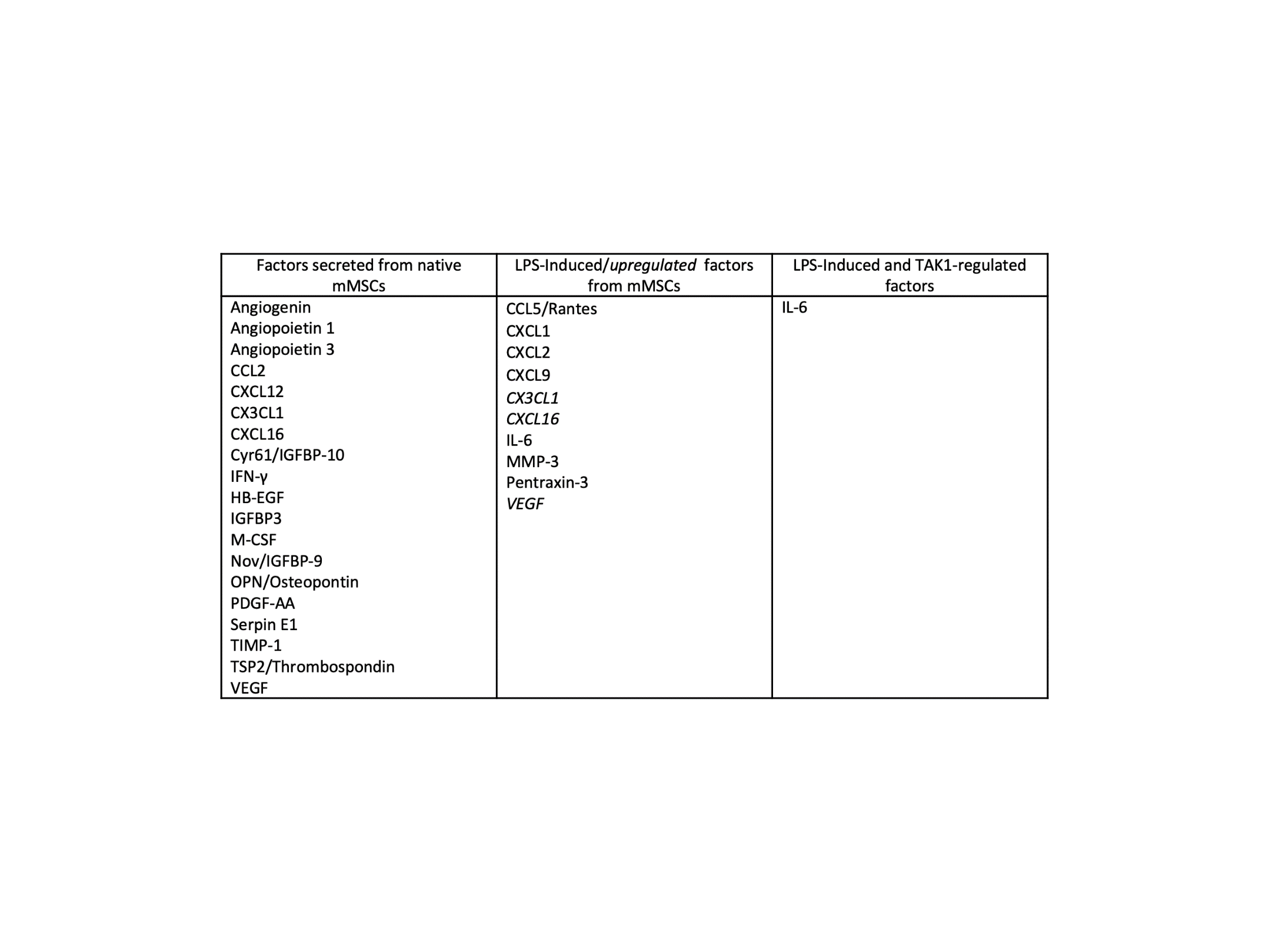
**

**Suppl. Fig. S3: Compilation of secreted factors from mMSCs detected by analyses of cytokine and angiogenesis arrays (R&D).** mMSCs secreted 26 factors in total, 10 of these were LPS-induced/upregulated. IL-6 only exhibited a substantial TAK1-dependent regulation.
